# Using multivariate cross correlations, Granger causality and graphical models to quantify spatiotemporal synchronization and causality between pest populations

**DOI:** 10.1186/s12898-016-0087-7

**Published:** 2016-08-05

**Authors:** Petros Damos

**Affiliations:** 1 Department of Environmental Conservation and Management, Faculty of Pure and Applied Sciences, Open University of Cyprus, Main OUC building: 33, Giannou Kranidioti Ave., Latsia, 2220 Nicosia, Cyprus; 2 WebScience, Mathematics Department, Faculty of Sciences, Aristotle University of Thessaloniki, University Campus, 59100 Thessaloniki, Greece; 3 Laboratory of Applied Zoology and Parasitology, Department of Crop Production (Field Crops and Ecology, Horticulture and Viticulture and Plant Protection), Faculty of Agriculture, Forestry and Natural Environment, University Campus, 59100 Thessaloniki, Greece

**Keywords:** Population modelling, Graph theory, MVAR models, Correlation and partial, Correlation, Final discovery rate, Synchronization, Causality

## Abstract

**Background:**

This work combines multivariate time series analysis and graph theory to detect synchronization and causality among certain ecological variables and to represent significant correlations via network projections. Four different statistical tools (cross-correlations, partial cross-correlations, Granger causality and partial Granger causality) utilized to quantify correlation strength and causality among biological entities. These indices correspond to different ways to estimate the relationships between different variables and to construct ecological networks using the variables as nodes and the indices as edges. Specifically, correlations and Granger causality indices introduce rules that define the associations (links) between the ecological variables (nodes). This approach is used for the first time to analyze time series of moth populations as well as temperature and relative humidity in order to detect spatiotemporal synchronization over an agricultural study area and to illustrate significant correlations and causality interactions via graphical models.

**Results:**

The networks resulting from the different approaches are trimmed and show how the network configurations are affected by each construction technique. The Granger statistical rules provide a simple test to determine whether one series (population) is caused by another series (i.e. environmental variable or other population) even when they are not correlated. In most cases, the statistical analysis and the related graphical models, revealed intra-specific links, a fact that may be linked to similarities in pest population life cycles and synchronizations. Graph theoretic landscape projections reveal that significant associations in the populations are not subject to landscape characteristics. Populations may be linked over great distances through physical features such as rivers and not only at adjacent locations in which significant interactions are more likely to appear. In some cases, incidental connections, with no ecological explanation, were also observed; however, this was expected because some of the statistical methods used to define non trivial associations show connections that cannot be interpreted phenomenologically.

**Conclusions:**

Incorporating multivariate causal interactions in a probabilistic sense comes closer to reality than doing *per se* binary theoretic constructs because the former conceptually incorporate the dynamics of all kinds of ecological variables within the network. The advantage of Granger rules over correlations is that Granger rules have dynamic features and provide an easy way to examine the dynamic causal relations of multiple time-series variables. The constructed networks may provide an intuitive, advantageous representation of multiple populations’ associations that can be realized within an agro-ecosystem. These relationships may be due to life cycle synchronizations, exposure to a shared climate or even more complicated ecological interactions such as moving behavior, dispersal patterns and host allocation. Moreover, they are useful for drawing inferences regarding pest population dynamics and their spatial management. Extending these models by including more variables should allow the exploration of intra and interspecies relationships in larger ecological systems, and the identification of specific population traits that might constrain their structures in larger areas.

**Electronic supplementary material:**

The online version of this article (doi:10.1186/s12898-016-0087-7) contains supplementary material, which is available to authorized users.

## Background

In recent years, there has been growing interest in graphical/causal models for the study of direct and indirect effects of climate on plant phenology and herbivores as well as the lagged effects of trophic or density depended factors on demographic parameters [1, 2]. Graphical models are a merger between probability and graph theory in which nodes represent variables and links non trivial interactions. Such constructs provide an important tool for facilitating communication among scientists, decision makers, and statisticians [3, 4]. Moreover, graphical models may be extremely important as an effective approach for describing multiple correlational associations and synchronization between ecological variables and coping with agricultural systems. In particular, problems in landscape ecology often involve modeling relationships among multiple physical and/or biological variables that may run on differing spatial scales [5]. However, although these problems are inherently multivariate, researchers commonly rely on univariate methods, such as autocorrelation functions and autoregressive and spatial regression models, to address them [6–9]. Moreover, the time dynamics and causality are often ignored, despite the fact that causality operates and corresponds to a mechanistic perspective of the function of the systems [10].

For insect populations in particular, two principal challenges are understanding the synchronization of insect population life cycles and identifying the causal agents of population progression rates. It is possible that species coexisting together in the same area exhibit synchronous population fluctuations because they are subjected to the same environmental conditions. However, it remains unclear whether there are any population similarities across sites and what specific mechanisms facilitate temporal synchrony or asynchrony in closely related species. Understanding the process of synchronization of dynamics is also a crucial aspect of understanding outbreak dynamics in population ecology, which allows the introduction of management activities and the mitigation of pest expansion [11, 12]. Nevertheless, discovering life cycle synchronization and causality requires statistical tools for separating the endogenous population dynamics from the effects of the time-dependent, and often correlated, forcing variables such as temperature [13–15]. From a statistical perspective, any coupling among ecological variables, also including abiotic drivers, is difficult to quantify and to distinguish from the endogenous correlation structures that are generated by the core ecological system (i.e. pest population).

Recently, networks or graphical models constructed from multivariate time series analysis based on correlations and causality measures have been extended to assess the existence of nonlinear dependences between several variables to offer a means of studying the interactions of complex biological systems [16–19]. Correlation is a normalized version of covariance that measures the linear relationship between serial data and is used to build a correlation network (after thresholding). In ecological studies, correlations tend to detect which populations (or variables in general) may be synchronized [19]. Furthermore, synchrony between populations can be described using cross-correlograms, which are graphs of lag correlations between series vs. lag intervals [20]. In addition, partial correlation measures apply to situations where the relationship between any two variables is influenced by their relationships with other variables. Nevertheless, one disadvantage of the above conventional approaches is that correlation does not mean causation [21, 22]. Correlated occurrences may be due to chance or even due to a common cause but are not necessarily connected by a cause–effect relationship. Thus, the introduction of causality rules may provide a robust means to distinguish whether any two ecological variables interact directly or whether the appearance of a correlation is a result of chance or the variables being forced by a common third variable.

Among the available measures of causality, Granger causality is probably the most popular. Granger causality is a statistical concept first proposed for deciding whether one time-series is useful in forecasting another [23]. Conceptually, Granger causality provides a much more stringent criterion for causation (or information flow) than simply observing high correlation with some lag-lead relationship. Therefore, the rule is particularly designed to address the estimation of causal connectivity to extract the features which characterize the underlying spatiotemporal dynamics rather than just modest correlations [18]. Granger received the 2003 Nobel Memorial Prize in Economic Sciences for applying such methods in stock markets. Since then, the method has been extended to include more variables to detect causality in very complex systems [24] and has been employed in econometrics (i.e., detection and forecasting of stock market interactions) [24, 25] and neuroscience (i.e., identifying directed functional causal interactions from time-series data) [26]. Recently, this method has been implemented in detecting causality in a complex ecosystem to initiate management policies [27] and for differentiating direct causal linkages from indirect causal linkages between multiple ecological state variables [28].

This work combines multivariate time series analysis and graph theory to detect synchronization and causality among certain ecological variables and to represent significant interactions via network projections. The main objective is to introduce sound statistical tools that are useful for the study of time-variant ecological processes and for describing potential interactions through graphical models. Particularly, four different approaches are used to describe multivariate interactions: simple correlations, partial correlations, Granger causality and partial Granger causality are used to describe multivariate interactions. These statistical measures correspond to different ways to compute the relationships between the different variables and to construct the networks using the variables (time-series) as nodes and the significant indices (correlations, Granger rules) as edges. The second objective aimed to apply the method to time series of pest populations in order to detect significant correlations and possible causation between the different variables. Moreover, how the different techniques can be used to build discrete graphical models and related network configurations is illustrated. Finally, efforts are made to compare the different network structures and to interpret some of the ecological processes such as the simultaneous emergence and seasonality of closely related insect species over an agricultural landscape.

The analysis of pest populations’ time-series data is of great interest for studying the driving parameters that explain pest population synchronization, and have practical implications for productively introducing time and location specific options for pest management. Moreover, using the proposed techniques to examine the synchrony of multi-species assemblages in ecology, may improve our understanding of how populations interact with long-term changes in their environments. This novel approach is shown to have advantages, not simply because it defines significant correlations among the variables but also because it may potentially capture some meaningful spatial relationships between ecological variables and related topological features. Moreover, conventional approaches do not directly address analyses of multiple ecological time series, while time-lagged causality (i.e., the difference in time units of a series of values and a previous one) is often neglected.

## Description of data

### Moth species

Three moth species were observed during 2003–2011: the peach twig borer *Anarsia lineatella* Zeller (Lepidoptera: Gelechiidae), the oriental fruit moth *Grapholita molesta* Busck (Lepidoptera: Tortricidae; previously known as *Cydia molesta*) and the summer fruit tortrix moth *Adoxophyes orana* (Fisher von Röslerstamm) (Lepidoptera: Tortricidae). The first-generation larvae of *A. lineatella* and *G. molesta* cause similar type of damage as they both attack young shoots, while *A. orana* is a leaf roller. During the farming season, larvae of later generations attack fruits in species-specific ways [29]. Altogether, the above Lepidoptera are widely distributed in Europe, North America and northern Asia and thus are viewed as the most important pests in stone fruit production worldwide [30]. Moreover, these species are polyvoltine and usually have 3–4 generations per year.

### Study area and population monitoring

**Fig. 1:**
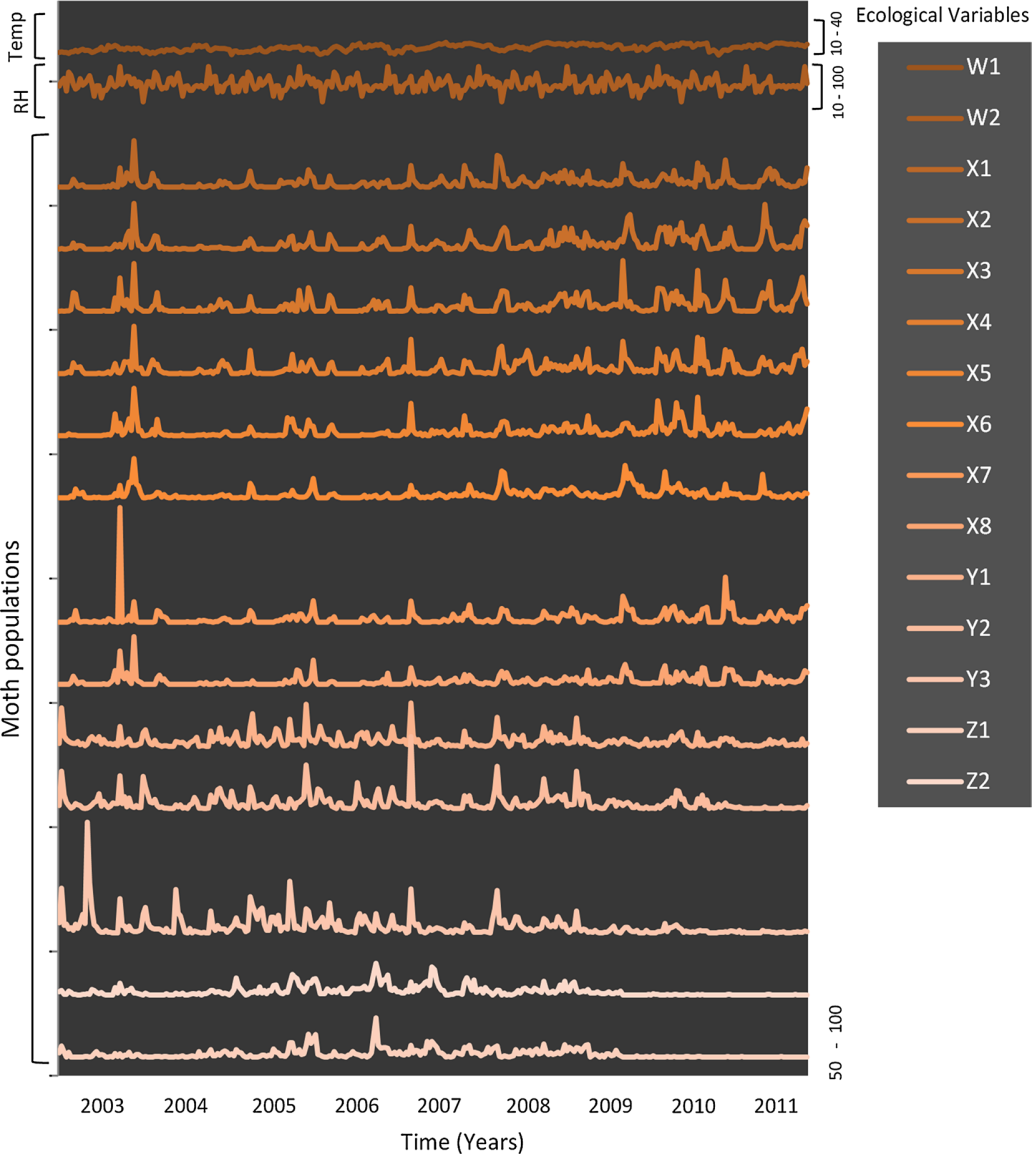
Actual ecological time series registered throughout the years 2003–2011 (3-day time intervals). W1: temperature (^o^C), W2: relative humidity (%), X1–X8: *A. orana*, Y1–Y3: *A. lineatella* and Z1–Z2: *G. molesta* moth populations (individuals)

Observations were carried out in a population sampling network that has been instituted in Northern Greece in particular in the prefecture of Imathia in Veroia (40.32 ^o^N, 022.18 ^o^E). The observation network covers an agricultural landscape that consists of plots in which fruit orchards represent approximately half of the observed field. The moth observation network consisted of 13 observation points. Trap placement sites were selected based on insect-host relationships (all included the main host, peach) and were representative in terms of cultivation conditions and landscape architecture. A cardboard delta trap (pheromone–pheromone traps: Trécé Inc., Salinas, CA, USA) were placed in each patch. Separate traps were used for each moth species, with sticky inserts baited with mixtures of synthetic sex pheromones (i.e., *A. orana*:(Z)-11-tetradecenyl acetate and (Z)-9-tetradecenyl acetate*, A. lineatella:* E)-5-decenyl acetate and (E)-5-decen-1-ol, *G. molesta*: (Z)-8-dodecenyl acetate) [31]. To avoid very strong autocorrelations, moths captured in traps were counted and removed twice a week, to create time series of the moth populations (Fig. 1, Additional file 1). Daily minimum and maximum air temperature data and relative humidity (RH) were obtained by using HOBO data-logging units (Onset Computer Corporation) placed on ALMME^®^ experimental fields and registered during the same period (2003–2011) [32]. All data were collected from field studies that complied with institutional, national and international guidelines.

These data were used to address the problem of transforming ecological time series into the correlation and causal networks given in the following section to propose statistical criteria to establish precise correlations and causal relationships.

## Methods for transforming ecological time series into a causal network

We consider a network, which consists of time-series which are represented as vertices (nodes) and are connected by edges (links) which are estimated through statistical indices. To construct the edges, which represent the link-interactions among the time-series, four different statistical methods were applied: cross correlations, partial cross correlations, Granger causality indices and partial Granger causality indices. Then, statistical significance tests and false discovery rates (FDR) are applicable to the outputs of the four techniques for trimming the networks. The four methods differ in the following ways: cross correlation is a simple measure of similarity of two series (say, X and Y) as a function of the lag of one relative to the other. Partial cross correlations correct the possible delayed effects of an additional variable (say, Z) on the correlation between X and Y. Granger causality provides a much more stringent criterion for causation (or information flow) than simply observing high correlation with some lag-lead relationship. Finally, partial Granger causality addresses the problem of eliminating the confounding influence of exogenous inputs and latent variables. In summary, the measures of correlation that were applied do not consider information from previous time steps (i.e., non-lagged correlation techniques) in contrast to Granger causality, which does considered it. Starting with the most simple and least conservative method, the different approaches are applied to examine how network configurations are affected and which best represents the final ecological relations. It is currently understood, for instance, that many ecological systems exhibit feedback, and it is therefore expected that cross correlations as a symmetric measure may be unsuitable for identifying nontrivial lag-lead relationships.

### Graph theoretic representation of cause-effect ecological network

To continue with the structure of graphs, based on multivariate time series analysis, it is convenient to introduce some graph theoretic matrix notations that mathematically formalize the networks proposed here. By definition, a graph *G* consists of a set of vertices-nodes (*V*(*G*) = {*υ*_1_, *υ*_2_, …, *υ*_*n*_}) and a set of edges-links (*E*(*G*) = {*e*_1_, *e*_2_, …, *e*_*n*_,}) in disjoint pair form, *G* = *(V, E).* Thus, the graph-network is an ordered pair [V (G), E (G)] [33, 34]. A graph is directed if the edge set is composed of ordered vertex (node) pairs and is undirected if the edge pair set is unordered. Any graph can be represented according to its adjacency matrix. The elements of the matrix indicate whether pairs of vertices are adjacent or not adjacent in the graph.

In the next sections, I intend to develop networks based on four different techniques (i.e., similarity measures) applied to compute the edges *e*_*ij*_ between any two nodes *v*_*i*_ and *v*_*j*_. Let ***E*** be an *nxn* matrix (*i* and *j* are indexes that go from 1 to n, and *e*_*ij*_ is a single entry of the matrix ***E***), called *similarity*-*values* matrix, that has as elements the similarity measure values (correlations, partial correlations, Granger causality, partial Granger Causality). Moreover, let ***E′***, or *pvalue* matrix, be a matrix with its elements being the significant probability values of multiple comparison test in respect to the similarity measure (correlations, partial correlations, Granger causality, partial Granger Causality) using either parametric or non-parametric comparisons, respectively. Thereafter, low probability values are considered to have more influence and to consist of the weighted versions of networks, which take into account the varying contributions of each causally significant interaction.

Lastly, an adjacency matrix, ***E″***, is considered for each similarity measure, for which the constituents’ *e*_*ij*_ are the outcomes of the final discovery rate:

1 $$e_{ij} = \left\{ {\begin{array}{*{20}c} 1 &\quad {if \,v_{i} v_{j} \in E} \\ 0 & \quad{ otherwise} \\ \end{array} } \right.$$

***E″*** is a *binary*-*adjacency* matrix and is used to record the most probable, non-trivial, numbers of edges joining pairs of vertices. Particularly, when the element *e*_*ij*_ of the matrix is one there is an edge from vertex *i* to vertex *j*, and when it is zero there is no edge.

Each matrix (*similarity*-*values*, *p*-*values* and *binary*-*adjacency*) contains the information about the connectivity structure of the graph and is further used for the extraction of information about the characteristics of the investigated ecological networks. Four types of networks were constructed according to the four different methods (cross correlations, partial correlations, Granger causality and conditional Granger causality) of calculating the elements of the matrix. The number of nodes was the number of the time-series variables (including both; biotic and abiotic variables). The *X*, *Y* and *Z* represent populations of *A. orana*, *A. lineatella* and *G. molesta*, respectively, while Temp. and *RH* represent the two abiotic variables: mean temperature and relative humidity.

### Standard measures of correlation for undirected graphs

#### Cross correlations

I look at the ecological time series, each of which is observed through successive seasons as a continuous univariate process: weekly counts are a vector that represents population realization available through observation. The Pearson correlation coefficient, *r*, is used as a standard similarity measure [35]:

2 $$cor\left( {X_{i} ;X_{j} } \right) = r_{{X_{i,} X_{j} }} = \frac{{s_{{X_{i,} X_{j} }} }}{{s_{{X_{i} }} s_{{X_{j} }} }}$$

where *s*_*XiXj*_ stands for the sample covariance of the variables *X*_*i*_ and *X*_*j*_ and where *s*_*Xi*_, *s*_*Xj*_ are the standard deviations of samples *X*_*i*_ and *X*_*j*_, respectively. Substituting the estimates of the covariance and variance based on a sample gives the Pearson’s linear correlation coefficient, with the following estimate:

3 $$e_{ij(cor)} = r_{{X_{i,} X_{j} }} = \frac{{\mathop \sum \nolimits_{t = 1}^{n} ({\rm X}_{i} - \bar{\rm X}_{i} ({\rm X}_{j} - \bar{\rm X}_{j} ) }}{{s_{{X_{i} }} s_{{X_{j} }} }}$$

Here, $\bar{\rm X}_{i}$ and $\bar{\rm X}_{j}$ are the sample means for the first and second variables, respectively,$s_{{X_{i} }}$ and $s_{{X_{j} }}$ are the standard deviations for each variable, and *n* is the series length (here, all successive years in which populations are active are considered) starting from t = 1.

### Partial correlations

The partial correlation network graphical representation, is defined as the collection of links between those nodes whose partial correlation (as defined below) is not zero. The linkage of these elements may be described in terms of an adjacency matrix that consists of a network with no direction (undirected) [31, 32]. To assess whether non-zero correlations are direct or indirect, causal measures should be considered as very useful because they measure the linear correlation between two variables after removing the effect of other variables and thus also finding spurious correlations and revealing hidden correlations. In particular, *cross correlations* are very suitable for detecting a type of dependence between pairs of variables (e.g., population *X*_*i*_ on population *X*_*j*_, vice versa, or both). However, because we include abiotic variables as well (e.g., *W*_*1*_: temperature and/or *W*_*2*_: relative humidity) it is most probable that both *X*_*i*_ and *X*_*j*_ are dependent on another variable *W*_*K*_ or even *m* other variables (nodes): *W*_*K*_ = {*W*_*k*1_, …, *W*_*km*_}, where *K* = {*k*_1_, …, *k*_*m*_}. Thus, we consider the above cases as trivial correlations that most likely do not suggest a link (*i, j*). To maintain ties among only the ecological variables with direct dependence the following partial correlation measure was introduced:

4 $$e_{ij(paco)} = \left. {\rho_{ij} } \right|W_{k} = \frac{{\left. {\sigma_{ij} } \right|W_{k} }}{{\left. {\sigma_{ii} } \right|W_{k} \left. {\sigma_{jj} } \right|W_{k} }}$$

where $\sigma_{{ij\left| {W_{k} } \right.}}$, $\sigma_{{ii\left| {W_{k} } \right.}}$ and $\sigma_{{jj\left| {W_{k} } \right.}}$ are components of a partial covariance matrix. The estimate of.*ρ*_*ij*_|*W*_*k*_ is the sample partial correlation *r*_*ij*_|*W*_*k*_ computationally derived as follows: First the residuals *e*_*i*_ and *e*_*j*_ of the multiple linear regression of *X*_*i*_ on *X*_*K*_ and *X*_*j*_ on *W*_*K*_, respectively, are computed. Next, the correlation coefficient of *e*_*i*_ and *e*_*j*_,.*r*_*ij*_|*W*_*k*_ = *r*_*ei*,*ej*_ is computed. Thus, if *X*_*i*_ (i.e., population of species *i*) and X_*j*_ (i.e., population of species *j*) are independent but, conditional to *W*_*k*_ (i.e., weather variable) then *ρ*_*ij*_|*W*_*k*_ should ideally be zero.

### Dynamic measures of correlation for directed graphs

In contrast to the rules for known correlations and partial correlation [32], the Granger-Causality approach proposed by Granger [23, 24] was also applied. One important asset, compared to non-lagged cross correlations, is that it provides a stream of interaction (i.e., directed cause—effect associations).

### Granger causality rules

The Granger causality measure, is based on the general concept of Norbert Wiener [36] that a causal influence should be manifest in improving the predictability of the driven process when the driving operation is followed. A measurable reduction in the unexplained variance of the response process (say, population *Y*_*i*_) as a result of inclusion of the causal (driving) process (say, population *X*_*i*_) in linear autoregressive modeling marks the existence of a causal influence from *X*_*i*_ to *Y*_*i*_ in the time domain *n* = 1, 2, … [37]. This method requires the estimation of multivariate vector autoregressions (MVAR) as follows:

5 $$Y_{t} = \mathop \sum \limits_{n = 1}^{p} a_{n} Y_{t - n} + \varepsilon_{r,t}$$

6 $$Y_{t} = \mathop \sum \limits_{n = 1}^{p} a_{n} Y_{t - n} + \mathop \sum \limits_{n = 1}^{p} \beta_{n} {\rm X}_{t - n} + \varepsilon_{u,t}$$

where *ε*_*r,t*_*and ε*_*u,t*_ are uncorrelated at the same time disturbances-residuals and *p* is the maximum number of lagged observations included in the autoregressive model. In addition, *α*_*n*_ and β_*n*_ are coefficients of the model (i.e., the contribution of each lagged variable to the predicted values of *Χ*_*i*_ and *Υ*_*i*_). The GCI is the pairwise linear Granger causality in the time domain and is defined as follows [18]:

7 $$e_{ij(GC)} = GCI_{X \to Y} = \ln \frac{{\sigma^{2} (\varepsilon_{r,t} )}}{{\sigma^{2} (\varepsilon_{u,t} )}}$$

where *σ*^2^(*ɛ*_*r*,*t*_) is the unexplained variance (prediction error covariance) of *Y*_*i*_ in its own autoregressive model (Eq. 5), whereas *σ*^2^(*ɛ*_*u*,*t*_) is its unexplained variance when a joint model for both *Y*_*i*_ and *X*_*i*_ is constructed (Eq. 6).

To provide useful heuristics for understanding the empirical ecological time-series, it is necessary to go beyond the simple two-variable vector autoregressive models and to study more variables that incorporate aspects of a more complex system. Thus, if ***X***_***k***_ = [*X*_1_*,X*_2_* ,…, X*_*k*_]^*T*^ denotes the realizations of *k* ecological variables and *T* denotes matrix transposition then the technique can be further extended by using the following multivariate vector linear autoregressive process (MVAR):

8 $$X_{t} = \mathop \sum \limits_{n = 1}^{p} A_{n} Y_{t - n} + E_{t}$$

Here *E*_*t*_ is a vector of multivariate zero-mean uncorrelated white noise process, A_*i*_ is the *k* × *k* matrices of model coefficients and *p* is the model order, chosen, in this case, based on the Akaike information criteria (AIC) for MVAR processes. Significant interactions are based on the standard Granger causality index (GCI). It is expected that GCI: *X*_*i*_* → Y*_*i*_ > 0 when *X*_*i*_ influences *Y*_*i*_, and that GCI: *X*_*i*_* → Y*_*i*_ = 0 when it does not. In practice, GCI: *X*_*i*_* → Y*_*i*_ is compared to a threshold value, and it was identified using parametric and non-parametric methods (i.e., using surrogate data).

### Conditional Granger causality rules

One disadvantage of the GCI is that indirect partial effects of the other variables are not touched (e.g., examining whether *X*_*1*_ Granger causes *X*_*2*_, by excluding the activities of all other variables *X*_*3*_*,…, X*_*i*_). This multivariate extension consists of the conditional Granger causality index (CGCI) and is extremely helpful because repeated pairwise analyses among multiple variables can sometimes give misleading results in terms of differentiating between direct and mediated causal influences [38].

As noted previously, the method requires the estimation of multivariate vector autoregressions (MVAR). If we consider *X* and *Y* as the driving and response systems, respectively, and conditioning on system *Z*, we have the following:

9 $$X_{t} = \mathop \sum \limits_{n = 1}^{p} a_{n} Y_{t - n} + \mathop \sum \limits_{n = 1}^{p} c_{n} {\rm Z}_{t - n} + \varepsilon_{r,t}$$

10 $$X_{t} = \mathop \sum \limits_{n = 1}^{p} a_{n} Y_{t - n} + \mathop \sum \limits_{n = 1}^{p} \beta_{n} {\rm X}_{t - n} + \mathop \sum \limits_{n = 1}^{p} c_{n} {\rm Z}_{t - n} + \varepsilon_{u,t}$$

The CGCI derived if we remove self-dependencies in respect to each variable in the Granger causality index for the two variables *X* and *Y* and conditioning on the third variable *Z* is as follows:

11 $$e_{ij(CGCI)} = CGCI_{X \to \left| Z \right.} = \ln \frac{{\sigma^{2} (\overset{\lower0.5em\hbox{$\smash{\scriptscriptstyle\frown}$}}{\varepsilon }_{r,t} )}}{{\sigma^{2} (\overset{\lower0.5em\hbox{$\smash{\scriptscriptstyle\frown}$}}{\varepsilon }_{u,t} )}}$$

where $$\sigma^{2} ( = \overset{\lower0.5em\hbox{$\smash{\scriptscriptstyle\frown}$}}{\varepsilon }_{r,t} )$$ and $$\sigma^{2} (\overset{\lower0.5em\hbox{$\smash{\scriptscriptstyle\frown}$}}{\varepsilon }_{u,t} )$$ are variances of error estimators of the above restricted and unrestricted vector autoregressive models [39]. The CGCI was also checked because it is highly useful in bringing out the causal interactions among sets of nodes by eliminating common input artifacts [26].

To remove noise from data and to produce robust estimates of temporal autocorrelations between successive dynamics, data were subjected to prewhitening prior causality analysis to reject as much as possible of the temporal autocorrelated white noise [40]. This must be done if, as is usually the case, an input series is autocorrelated and to avoid having the direct cross-correlation function between the input and response series yield a misleading indication of the relation between the input and response series in an autoregressive moving average (ARIMA) modeling process. This task was performed using the standard MatLab prewhitening filter.

### Significance of similarity measures and network trimming

The correlation coefficients between the variables form a correlation matrix that represents a weighted network. However, to detect only significant interactions and to trim the networks, it is important to apply a statistical hypothesis test according to a predefined probability that serves as the threshold level. This can be accomplished either by parametric or non-parametric methods, as indicated below.

### Parametric test of correlation

To reduce the possibility that the observed correlations occurred by chance, a significance test should be performed. Here the null hypothesis *H*_0_ that no correlations between any two ecological time series was examined, under the alternative hypothesis of the existence of correlations (two-tailed test). Thus if *ρ*_*ij*_ is the true Pearson correlation coefficient, then the hypothesis test for significance is *H*_0_:*ρ*_*ij*_ = 0, with the alternative *H*_1_:*ρ*_*ij*_ ≠ 0 and the estimate for *ρ*_*ij*_:*r*_*ij*_ = *s*_*ij*_*/s*_*i*_*s*_*j*_. Thus, if we take for granted that each paired data set is normally distributed and stationary, then the paired variables *X*_i_ and *X*_*j*_ yield.

12 $$(X_{i} ,X_{j} ) \cong N([\mu_{i} ,\mu_{j} ],[\sigma_{i}^{2} ,\sigma_{j}^{2} ],\rho_{ij} )$$

where *μ* and *σ*^*2*^ are the standard notations for mean and variance, respectively. The *t* statistic for a sample *n* is

13 $$t = \frac{{r_{ij} \sqrt {n - 2} }}{{\sqrt {1 - r_{ij}^{2} } }},$$

where *r*_*ij*_ is the correlation coefficient and *H*_0_ was rejected if: |*t*| > *t*_*N*−2; *a*/2_ for the *α* = 0.05 significance level.

For each pairwise tests the *p* values represented the probability of error that was involved in accepting the observed correlations of the ecological time series as valid. If the *p* values of the test were smaller than the *α*-threshold level (0.05), then the correlations were considered as significantly different from zero and a connection was traced in order to construct the ecological time series network.

### Non-parametric testing of correlation

Although ecological time series in this work are treated as stationary, bootstrapped randomizations for 100 surrogates were carried out, and non-parametric comparisons were also performed for confirmative reasons. In particular, this method should be applied in cases of small sample size and absence of information concerning deviation from normality and stationarity. Here, we derive the null distribution of *ρ*_*ij*_ from resampled pairs consistent with *H*_0_: *ρ*_*ij*_ = 0. Considering the original pair of ecological time-series (*x*_*i*_, *x*_*j*_), we generated B randomized pairs (*x*_i_^*b^, *x*_j_^*b^), *b* = 1, …, *B*. Although this random sample permutation destroys the time order, it uses the same distribution as the original time-series. At a next step, the *r*_ij_^*b^on each pair (*x*_i_^*b^, *x*_j_^*b^) and the ensemble $\left\{ {r_{ij}^{*b} } \right\}_{b = 1}^{B}$ that forms the empirical null distribution of *r*_*ij*_ were computed. *H*_*0*_ was rejected if the sample *r*_*ij*_ was not in the distribution of $\left\{ {r_{ij}^{*b} } \right\}_{b = 1}^{B}$. The null hypothesis was *H*_0_:*ρ*_*ij*_|*K* = 0 and the alternative *H*_1_:*ρ*_*ij*_|*K* ≠ 0. The analysis was carried out using the MatLab procedure; in all cases randomizations for 100 surrogates were performed.

### Significance test for GCI and CGCI

If the variable *X* does not Granger cause *Y* then the contribution of *X*-lags in the unrestricted model (Eqs. 5 and 9) should be insignificant, and the model parameters should therefore be insignificant. The null hypothesis is *H*_*0*_: *b*_*i*_ = 0, for all *i* = 1, *…, p* and the alternative is *H*_1_: *b*_*i*_ ≠ 0, for any of *i* =  1,* …, p*. A rejection of the null hypothesis implies that there is Granger causality and this can be evaluated using the *F*-test (Snedocor-Fisher) [41]:

14 $$F = \frac{{(SSE^{r} - SSE^{u} )/p}}{{SSE^{u} /ndf}}.$$

Here, SEE is the sum of square errors, *ndf* = *(n*−*p)*−2*p* is the degrees of freedom, *n*−*p* is the number of equations, and 2*p* is the number of coefficients in the unrestricted model (Eqs. 6 and 10).

### False discovery rates and true correlations

The false discovery rate (FDR) multiple testing procedure was applied to correct the false significant correlations of multiple comparisons, which can arise from incorrect rejections of false positives. The FDR was applied for conceptualizing the rate of *type I* errors in null hypothesis testing when conducting the multiple comparisons of the series and for further trimming the networks. The FDR was suggested by Benjamin and Hochberg [42] to address the problem with performing multiple simultaneous hypothesis tests. The FDR is a powerful concept by which one can retain the statistical power that would be lost to simultaneous comparisons made with Bonferroni-type procedures.

In particular, as the number of hypotheses increases, so does the probability of wrongly rejecting a null hypothesis because of random chance [42]. Therefore, to correct for multiple testing, as the same test applies for any *i* and *j* in {1, …, *n*}, one can use the correction of the false discovery rate (FDR) [43]. According to the procedure, first the *p*-values of *m* = *n*(*n*−1)/2 tests are set in ascending order *p*(1) ≤ *p*(2)… ≤ *p*(*m*). Next, the rejection of the null hypothesis of zero correlation, at the significance level *α*, is decided for all variable pairs for which the *p*-value of the corresponding test is less than *p*(*k*), where *p*(*k*) is the largest *p*-value for which *p*(*k*) ≤ *k*·*α*/*m* holds [44].

## Results

### Weighted networks using cross and partial cross correlation

**Fig. 2:**
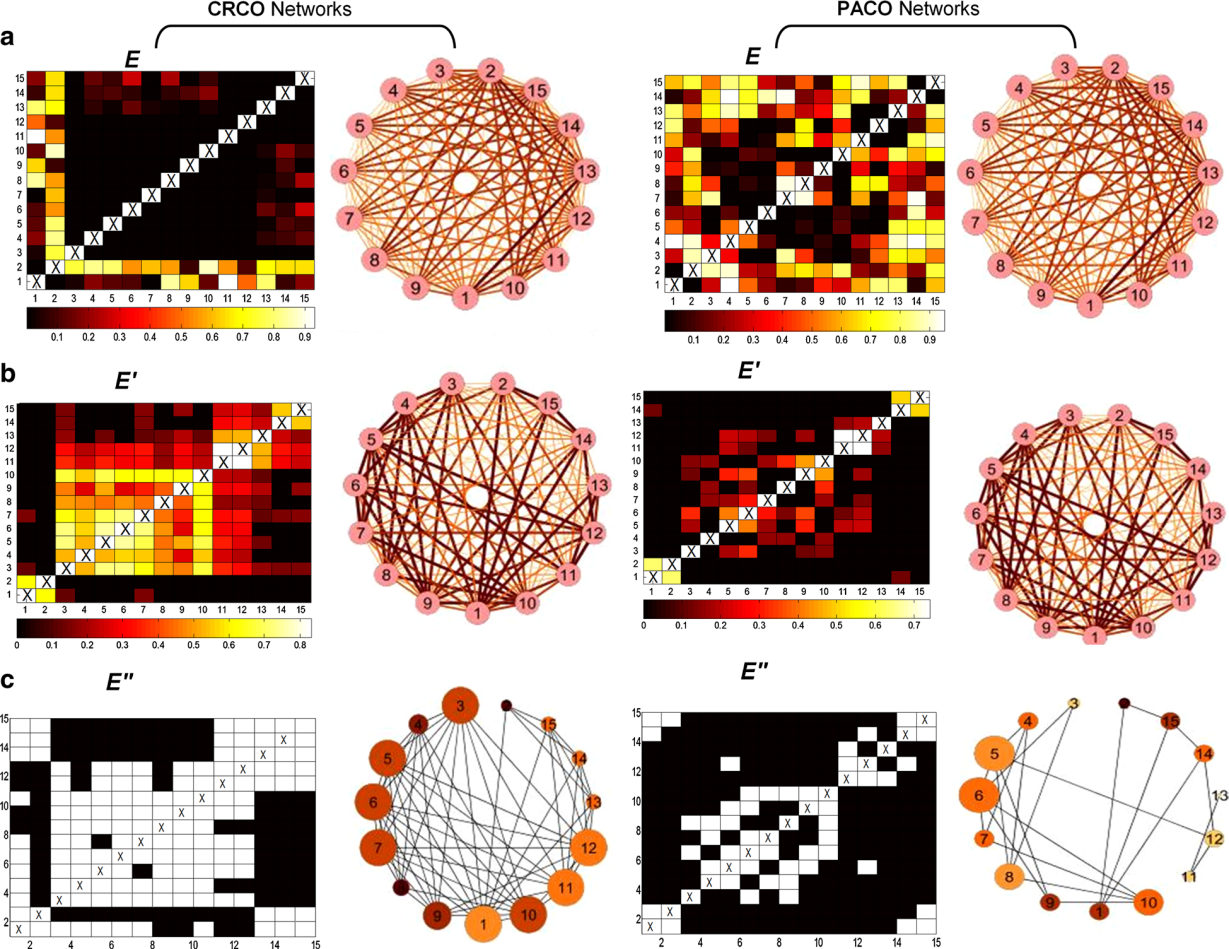
Cross correlation (*CRCO*) and partial cross correlation (*PACO*) statistical similarity measure matrices and the respective network configurations; **a** *similarity*-*values* matrices (***E***) and related full connected weighted networks: computed using multivariate cross correlations and partial cross correlations. **b** Significant *p*- *values* matrices (***E’***) and related full connected weighted networks: computed using multivariate tests of correlation using the *α* = *0.05* significance level for thresholding and **c** *binary*-*adjacency* matrices (***E’’***) and related binary networks: computed using false discovery rate (*FDR*) to correct and adjust false *p* values. Notice that the *binary*-*adjacency* matrix is composed of an upper and *lower triangular part* which are identical in which the white cells indicate significant interaction between the time-series and are used to draw the links. Matrix corresponds to K = 15 ecological time series of length n = 285 (intervals of 3 Julian day during the growth season; W1: temperature (^o^C), W2: relative humidity (%), X1–X8: *A. orana*, Y1–Y3: *A. lineatella* and Z1–Z2: *G. molesta* moth population (individuals). Deeper (hot) colors, in either matrices or networks, indicate higher correlations and implications for each pair of variables tested, while *light colors* and thinner vertices are connected to lower correlations and implications

Successive captures of moth traps throughout the observation period and weather data are presented in Fig. 1. Figure 2a depicts the *similarity*-*values* matrices ***E*** (i.e., significant measure values, whereas non-significant values are set to zero) and the related weighted networks, showing each with the statistical similarity measures that were applied to construct them, i.e., the cross correlation networks (CRCO) and the partial correlation networks (PACO). Figure 2b depict the significant *p*-*values* matrix (***E***′) (left) and the resulting weighted networks for the CRCO and PACO similarity measures (right). These graphical depictions provide a first model of a significant interaction flow based on their correlation. The structure of these weighted networks was established using the cut-off threshold *α* = 0.05. Figure 2c presents the *binary*-*adjacency* matrices (***E***″) and the related network configurations obtained after the final discovery rate analysis (FDR). These are binary values (0 or 1), and the associated network is therefore not weighted. However, vertices having higher degrees and clustering coefficients are represented as larger nodes and with darker colors, respectively.

Because the CRCO and PACO-FDR networks represent only the significant correlations among the variables, some very interesting information can be identified. In particular, in the PACO-FDR networks, significant correlations are generally observed among populations of the same species.

In addition, the weather variables, temperature and relative humidity, are equally interlinked. For example, we discover that the nodes Y11, Y12 and Y13, which represent populations of *A*. *lineatella*, form a triangle. The nodes Z14-15, which correspond to populations of *G. molesta*, are connected, whilst populations of *A. orana*, nodes X3, X4, X5, X6, X7, X8, X9 and X10, define a subgraph. Eventually, the two weather variables are also connected, a pattern that again is clearer when observing the PACO-FDR networks because the components of the weighted networks are fully linked.

### Granger related binary causal networks

**Fig. 3:**
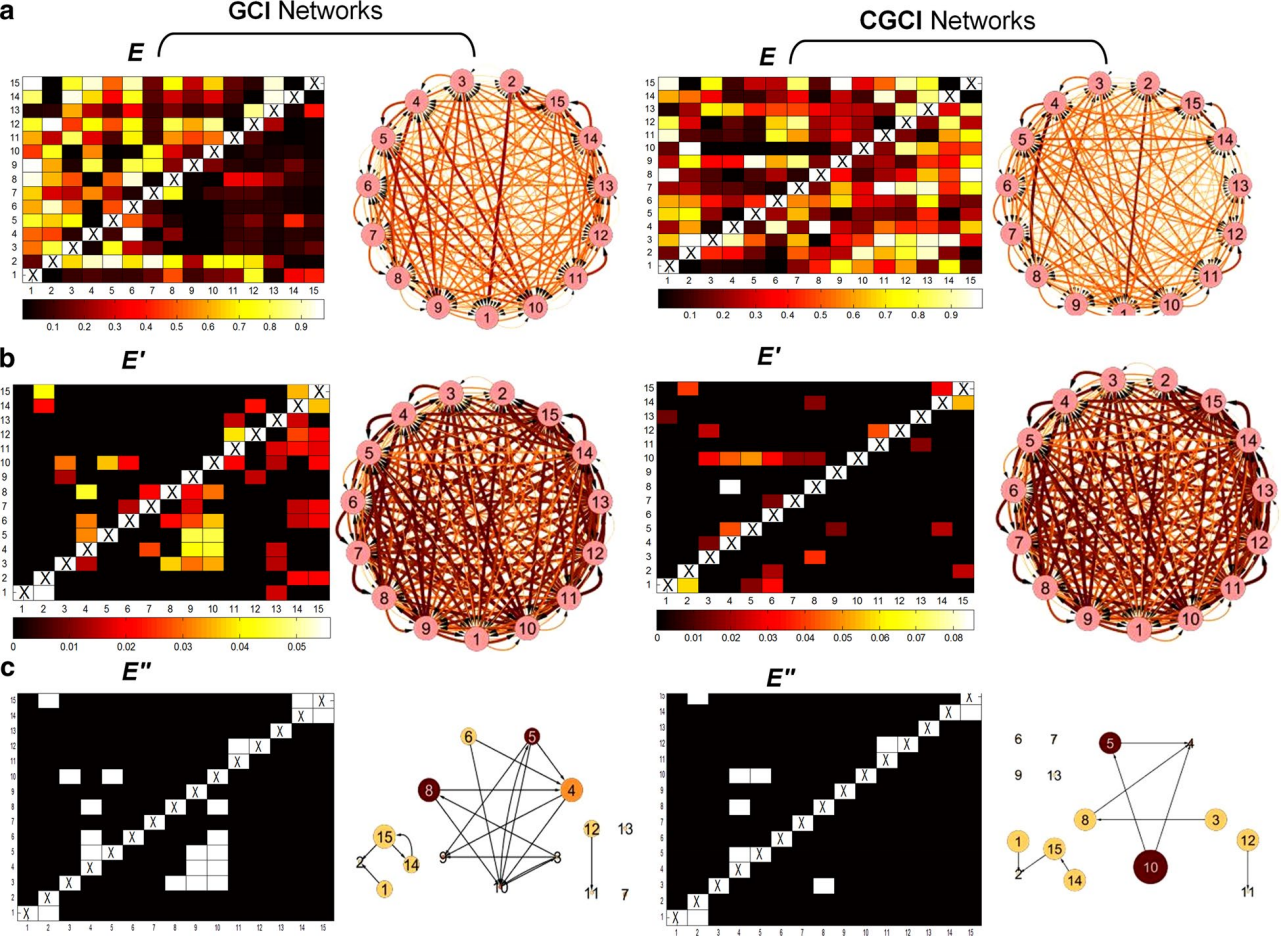
Granger causality (*GCI*) and conditional Granger causality (*CGCI*) similarity measure matrices and the respective network configurations; **a** Granger *similarity*-*values* matrices (***E***) and respective full connected weighted networks: computed using Granger causality index and conditional Granger causality index. **b** Significant *p* *values* matrices (***E’***) and related full connected weighted networks: computed using multivariate parametric tests using *α* = *0.05* significant level for thresholding and **c** *binary*-*adjacency* matrices and related binary networks: computed using false discovery rate (*FDR*) to correct and adjust false *p* values. Notice that the *binary*-*adjacency* matrix is composed of an *upper* and *lower* *triangular part* which are non-identical, therefore, the white cells, indicating significant interactions between the time series include also the direction of causality which was used to draw the links. Matrix corresponds to K = 15 ecological time series of length n = 285 (intervals of 3 Julian day during the growth season; W1: temperature (^o^C), W2: relative humidity (%), X1–X8: *A. orana*, Y1–Y3: *A.* *lineatella* and Z1–Z2: *G. molesta* moth population (individuals). Deeper (hot) colors, in either matrices or networks, indicate higher correlations and implications for each pair of variables tested, while *light colors* and thinner vertices are connected to lower correlations and implications

Figure 3 shows the adjacency matrices (left) and the respective directed causal networks (right) constructed based on the Granger causality (GCI) and the conditional Granger causality index (CGCI).

In particular, Fig. 3a depicts the *similarity*-*values* matrices (***E***) with the significant measure values of the GCI and the CGCI, while Fig. 3b displays the *pvalue* matrices (***E’***) after the pairwise multivariate analysis and the parametric hypothesis testing. The GCI and CGCI causal networks are directed, and their matrices are therefore not symmetrical, in contrast with Fig. 2. This is associated with the fact that Granger measures produce non-symmetric adjacency matrices; thus, the associated networks are able to designate the direction of causalities. Moreover, the GCI and CGCI causal networks display a less dense form than the CRCO and PACO networks and remove the links between species and the Granger rules, thereby showing which of the correlated variables are cointegrated and share common stochastic drift. This represents an advantage of the Granger causality measures compared to simple non-laged cross correlations.

Furthermore, and as noted above, to avoid the multiple testing problems, p values were estimated using the false discovery rate as shown in the *binary*-*adjacency matrices* (***E***″). In Fig. 3c, larger nodes represent variables having higher degrees, and deeper colors represent higher clustering coefficients. Thus, variables 5 and 10, which correspond to populations of *A. orana*, have higher out-degrees and clustering coefficients. From a graph-theoretic standpoint, these nodes can be viewed as hubs. A hub contains multiple links and is of exceptional interest for any network configuration. Moreover, based on the CGCI-FDR networks, at least two subgraphs are observable. The first consists of populations of *A. orana*, while the second includes variables 14 and 15, which both belong to the species *G.molesta*. Moreover, the two abiotic variables, nodes 1 and 2, are related.

### Force-directed causal network configurations

Force-directed network layouts are constructed based on forces assigned to the set of edges and nodes to obtain interpretable community structures in multipartite networks. Here, I have used the default Barnes–Hut approximation algorithm in Cytoscape [45].

**Fig. 4:**
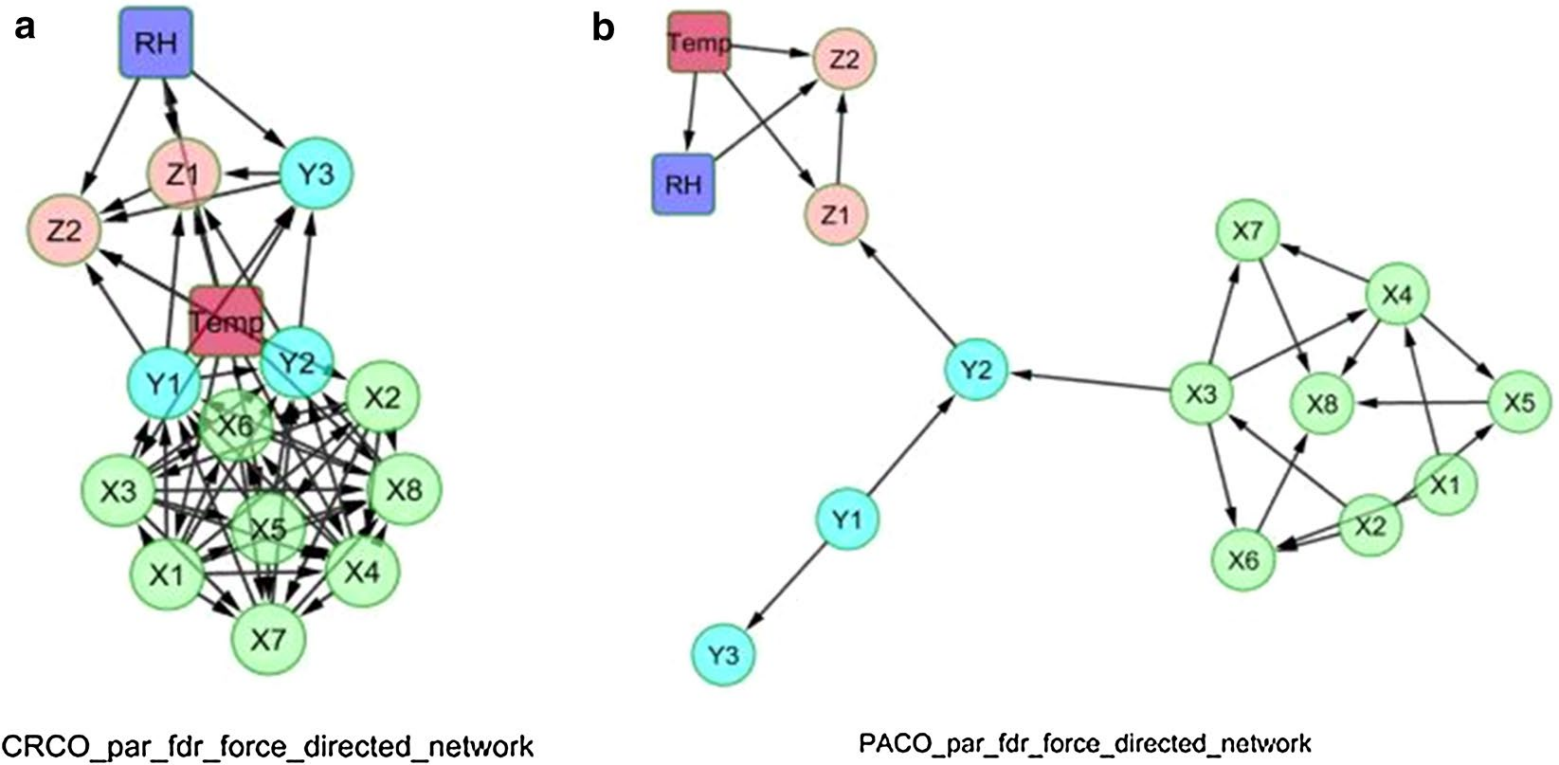
**a** Force directed network layouts for cross correlations (*CRCO*) and **b** partial cross correlations (*PACO*) and after the false discovery rate (*FDR*) correction that was used to calculate their adjacency matrix. The networks are based on assigning forces among the set of links and nodes to obtain interpretable community structures using the Barnes–Hut approximation algorithm of Cyto-scape (Xi: *A. orana*, Yi: *A. lineatella* and Zi: *G. molesta*; Temp: mean temperature; *RH* relative humidity)

**Fig. 5:**
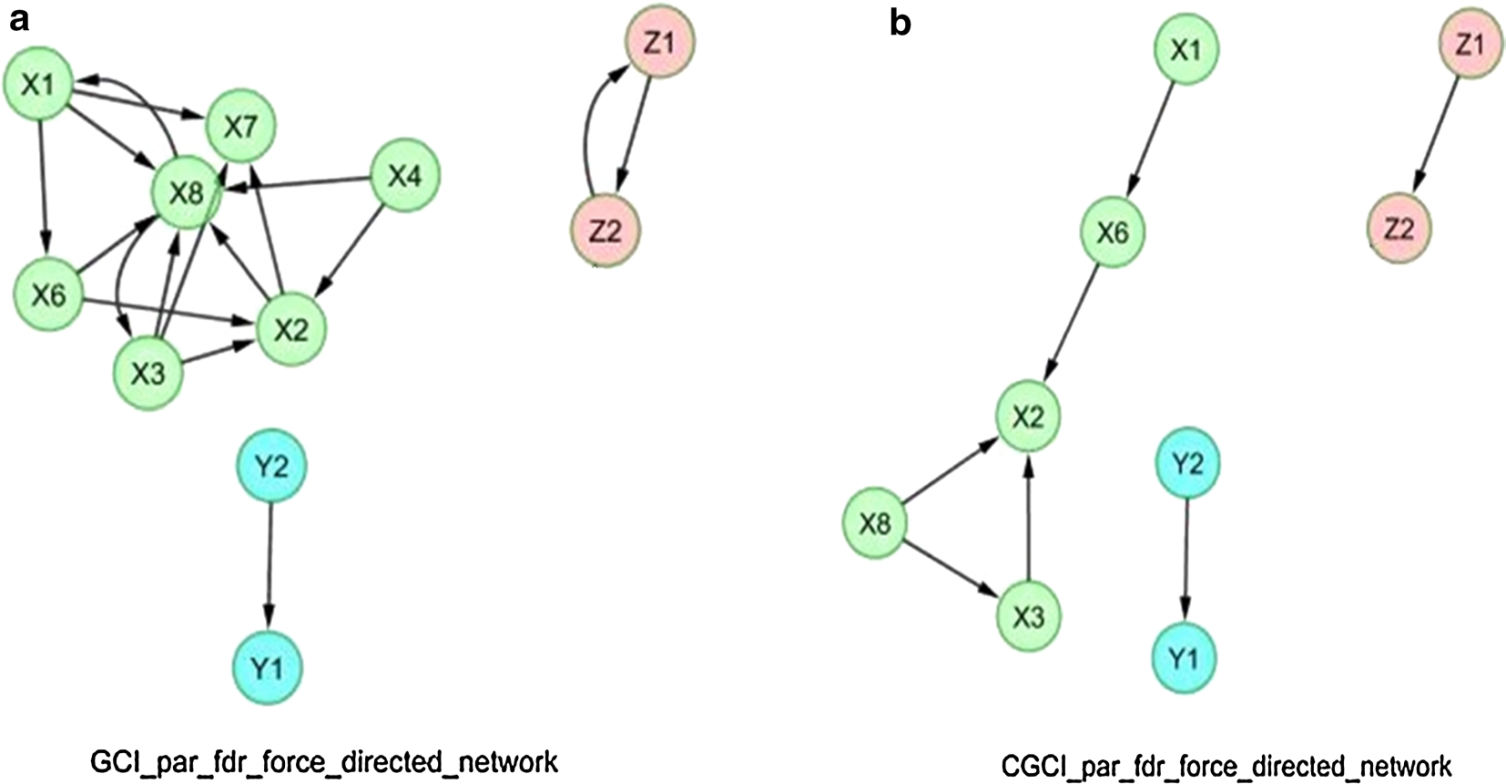
**a** Force directed network layout for Granger causality and **b** causal Granger causality index (*GCI* and *CGCI*) parametric analysis after the false discovery rate (*FDR*) correction that was used to calculate their adjacency matrix. These particular networks are based on assigning forces among the set of edges and links and after removing non-incidental connections to obtain interpretable community structures using the Barnes–Hut approximation algorithm in Cyto-scape Xi: *A. orana*, Yi: *A. lineatella* and Zi: *G. molesta*; Temp: mean temperature; *RH* relative humidity)

Figures 4 and 5 depict the force-directed network layouts that correspond to the matching networks given in Figs. 2c and 3c. However, the GCI and CGCI networks of Fig. 5 have been forced to show only non-incidental connections. Based on these configurations, the interaction patterns of the biological variables are more easily indicated. All partial configurations clearly show the presence of sub graphs that consist of populations belonging to the same species, and they enhance the fact that Granger rules provide a robust method for removing trivial links and trimming the networks.

The weather variables have no significant driving role in most cases and especially in the case of the partial correlation analysis. This attribute probably suggests that the effect of environmental variables is diminished after the current network analysis, although these variables (especially temperature) generally have a substantial impact on insect population ecology [30]. However, in some cases, incidental connections have also appeared. This oxymoronic result was not unexpected, considering that the methods that were applied to introduce links are strictly statistical in nature and not based on biology.

### Graph-theoretic and landscape-related network projections

**Fig. 6:**
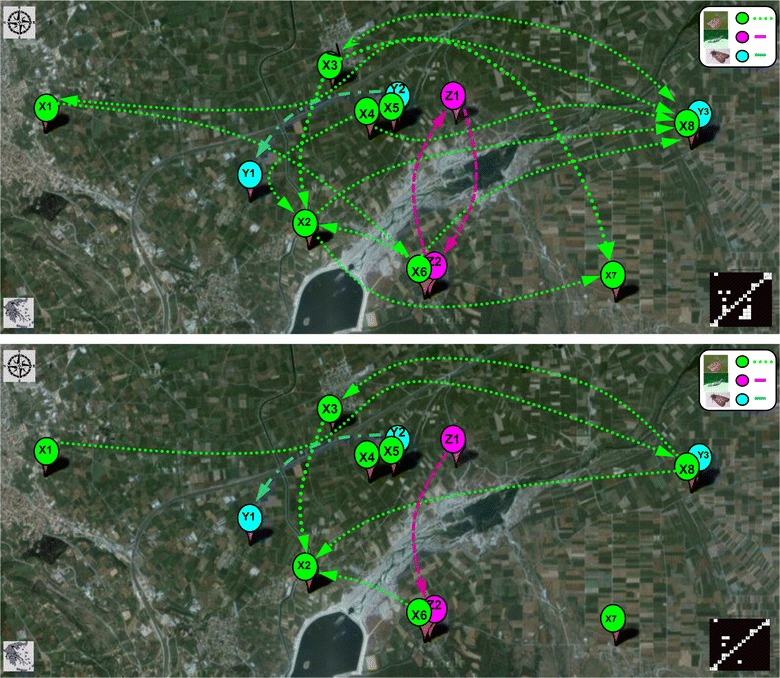
Google maps layer of the observation region and related Landscape projections of causal population networks, CGI-FDR network (*upper picture*) and CGCI-FDR network (*lower picture*), with respect to sampling points and region-specific topology (scale: 1:5000; area: A~8 × 16 km^2^; Xi: *A. orana*, Yi: *A. lineatella* and Zi: *G. molesta*; weather variables are not included)

Because the GCI and CGCI networks are directed, they are the only networks that have been overlaid on the observation region to identify the location of the causal forcing variables. Figure 6 depicts the topological projections of the GCI-FDR and CGCI-FDR causality networks over the agricultural landscape. Each node represents a site where moth populations were observed, while the arrows represent the Granger causal relations. A higher number of interactions is observed in the GCI network (upper picture) than in the CGCI-FDR causality network (lower picture). This happens because prior to the projection, indirect preferential effects of any other variable were removed based on the false discovery rate procedure. The observation of significant interspecies associations was dependent on the type of analysis. Moreover, based on the topological projections of Fig. 6, it is apparent that some nodes, which represent landscape-related population activity, point to some particular locations and not το all sites where observation was performed. For instance, in the CGCI-FDR network, it is apparent that most populations of *A. orana* and *A. lineatella* of the West side point to those of the east side. Furthermore, Fig. 6 more clearly indicates the landscape-related locations where populations act as hubs and may function as ‘hot spots’ of significant population interactions. Based on the landscape properties, the experimental area can be split into two major areas, northwest and southeast, separated by a river (Aliakmon). Based on the current analysis in these areas, the intra-species interactions seem to act across the physical river border and not just inside each sub-region. The GCI network shows 10 maximum relations across the river and 4 within sub-regions relations, while the CGCI network shows 5 relations across the river and only 2 within sub-regions. However, this may have resulted from several factors, including the inherent properties of a species population in relation to environmental conditions [46, 47]. Warmer temperatures and increased precipitation, for instance, can reduce the rates of population growth [48]. Additionally, because there is no similar analysis in the literature, some of the answers are difficult to translate from a strictly agro-ecological perspective. Nevertheless, the results provide partial support to the hypothesis that moth population emergence in one location is synchronized with that of populations located nearby, that emerge a few days later, and this dynamic can be described via the landscape-projected cause-effect graphical models.

## Discussion

In this work, the question of evaluating significant relationships between ecological time-series has been addressed based on multivariate time-series analysis and graph-theoretical approaches. In particular, spatial associations among a set of ecological entities have been studied, including two abiotic variables and thirteen biotic (moth population) variables. The most recent statistical measures have been adopted to assess the significances of correlations and to construct causal networks, which may provide information on landscape-related species synchronization and causation.

The detection of spatial synchrony is of great ecological interest since it provides answers on which factors affect the observed patterns of spatial population synchrony. For example, although a positive environmental correlation with populations across sites has been observed in both, the similarity-values networks and probability-values networks its effects degenerate in the binary-adjacency networks. These results indicate not only that environmental correlation and population spatial dynamics should be looked at in combination, but also that we may derive to different conclusion depending on the rigor of statistical criteria used to define non trivial relations. Additionally, because correlation does not imply causation way may obtain different interpretation when using causality measures over simple correlations. Therefore, causality networks not only show, which population may be spatial synchronized, but provide some evidence of the causes of synchrony of interconnected populations (i.e. growth, dispersal or noise).

The current network construction approach differs to that of most ecological network analyses, because it offers both heuristics and practical methods to define the strength of associations among the ecological variables of interest. The core idea is to integrate knowledge of each population time series in the description and interpretation of all others. These data are then used to determine the order of the multivariate autoregressive model (MVAR) used for the correlation similarity and Granger causality measures. By removing the correlations inherent to the time-series, as well as the partial series of multivariate interactions, we derived the final network configurations. We conclude that although the constructed networks do not come in any ecological category in a narrow sense, traditional population community studies and all ecosystem approaches (which additionally emphasis on energy influxes, including biomass and nutrient cycles [49, 50]), they may provide a new qualitative method for building and studying complex ecological network organizations.

Furthermore, in contrast to other studies based on correlations *per se*, the current approach differs in two ways. First, it defines an a posteriori correlation between ecological variables to detect synchronization, using statistical hypothesis testing; second, it provides a means to discover the existence of causal dependences between the different variables. One additional advantage is that all four methods of constructing networks can be implemented in the absence of any restrictive presuppositions regarding the underlying dynamical structure of the data set. Therefore, the use of the current multivariate techniques may contribute to detecting population synchronizations as well as the relative importance of biotic and abiotic processes to the distribution of population abundance and its dynamics.

From an ecological standpoint although synchronized fluctuation in abundance among spatially segregated populations and ecological variables is common in nature, the identification of the relative contribution of each factor to patterns of synchrony remains a challenge [51, 52]. Furthermore, when the processes that contribute to synchrony are studied in isolation, the synchrony patterns can be ascribed to the underlying cause [53]. However, under field conditions, ecological systems are more complicated, with intrinsic and extrinsic causal processes appearing together [54] and therefore, the proposed method represents a robust approach for detecting multivariate spatial synchronization and causality between ecological time-series.

Moreover, based on the current case study results, we can extract some very interesting ecological information. For example, these results show that although the environmental variables are to a high degree correlated with the populations, they are not acting as the main driving causal forces. Therefore, the results support the hypothesis that synchrony and causality can also be induced by other factors (i.g., local dispersal among populations, competition, host allocation and many more). Furthermore, these results show that spatial synchrony and causation are more likely between populations of the same species.

However, it is also important to note that the transformation of connectivity values from a continuous to a binary scale generally entails difficulties and has some drawbacks. In particular, while the binary scale clearly enhances contrast, it also hides potentially vital information as connectivity values move below or above threshold levels. Still, the general appearance of the weighted population graphs is not qualitatively very different from the binary ones. In reality, very similar connectivity patterns are replicated in both graph categories (i.e., the circular as well as the force-directed layout).

Comparing the basic methods that have been applied to construct the networks (Correlations and Granger rules of connectivity) it is important to note that each method may provide a different range of interpretation. Although both methods may account for synchronization, correlation shows which variables are synchronized but not which drives the others as in the case of the Granger rules. Moreover, the advantage of the Granger statistical rules is the incorporation of a simple test to determine whether one series (population) is being caused by another series (i.e., environmental variable or nearby population) even when they are not correlated. Therefore, the Granger method is particularly suitable for estimating the directed connectivity of time-series data in order to extract features that characterize the underlying spatiotemporal dynamics. For example, the landscape-related causality networks are used for studying the relationship between simultaneously recorded populations and provide insight into which population-locations act as driving variables (i.e., hubs) and may used to predict an increase or decrease in linked population locations.

Moreover, partial correlation and partial causality measures are able to remove the correlation between any two variables that is present just because they are correlated with a third variable [55, 56]. For instance, the fact that two different species are each correlated to temperature does not mean that they are also related to each other. Finally, the FDR networks also provide the most robust network construction in terms of statistical power. The FDR approach is a new statistical procedure, and this technique is even more valuable for cases in which multiple calculations are performed on large-scale data sets. Moreover, the key advantage of FDR is that it takes into account a priori fast control of the mean fraction of the false rejections made over the total number of rejections performed and thus avoids bias. Actually, the FDR procedure is quick and easy to compute and can be trivially adapted to work with correlated data as well [57]. Consequently, if we mean to choose among the methods applied to build the networks, those using partial correlation measures are more reliable because they take out the effect of the random variables which may create links just by chance. Moreover, this study brings out the significance of the false discovery rate to capture only the real non-trivial links to generate accurate and informative networks. The usefulness of the FDR approach is all-important as the number of time series increases.

Despite the promising outcomes of the current analysis, there are also some restrictions. First, the case subject field was studied based on a relatively modest percentage of the population and biotic components; thus, the interpretation of causal influence is determined solely by the measured variables. We cannot exclude, for instance, potential modification of the results if additional variables are included. For example, as the network size is changed (i.e., by adding more populations variables and species), it may be possible that new edges will appear even if the underlying network topology remains identical [58, 59]. Thus, some caution must still be exercised when considering overall patterns in the moth population network structures, because they rely upon a relatively small number of variables observed in that particular area of research. Therefore, we should consider that the current network construction method should be regarded more as a method for the investigation of population synchronizations and causality between discrete variables rather than as a way to understand actual interactions between species. Only assumptions can be made regarding whether the same relationships also hold in other areas despite several other factors that affect actual population dynamics and are that are not considered in the present study; therefore, the ecological inference remains somewhat rudimentary.

From a stricter ecological standpoint, therefore, it is rather difficult to interpret why moth populations are mostly correlated along an east–west axis through physical barriers rather than into nearby regions within the same habitat. One hypothesis is that because the ‘driving population variables’ benefit from local micro environmental conditions, the moths emerge a few days earlier and cause a lagged emergency in the adjacent population. There are no similar studies that would allow a direct comparison, but for poikilothermic organisms and insects in particular, moderate temperatures and rich humidity favor reproduction and development [15]. In closely related species such as the codling moth *Cydia pomonella*, geostatistical spatial analysis revealed that high populations are captured at sites most suitable for the pest and the host [60]. Moreover, similar spatial analysis performed in South Italy for *A. lineatella* and *G. molesta* showed that the main ‘hot spot’ for both lepidopterous pests was in a stone fruit orchard in the northern zone of the study area; other infested areas were in peach orchards and, in the case of *A. lineatella*, also in plum orchards. However, in contrast to the current study, that analysis showed a river acting more as a barrier than as an ecological corridor [61]. Still, it is noteworthy to say that the current analysis differs from the affront mentioned geostatistical approaches in terms of their objectives, implementation and interpretation of the outcomes, and especially because they are using probabilistic models based on the spatial domain without reference to time. The Granger rules provide attention to extrapolations for making forecasts, and although geostatistics may be interested in extrapolation, the methods are optimized for interpolation. Hence, because there are no related subjects using the current approach, comparison of the current results on an ecological-behavioral basis is rather difficult. Therefore, the study additionally emphasizes that the new methodological approach can be used to develop ecological networks and does not provide strict ecological answers regarding the function of the study system.

In summation, the constructed networks may provide an intuitive, advantageous representation of the relationships among multiple pest population that may be present within the agro-ecosystem. For example, the present work not only validates the hypothesis that populations belonging to the same species are correlated, by showing synchronized population dynamics, but also that some of them may have prominent roles in population dynamics as driving variables. For pest control, the incorporation of spatial information into an Integrated Pest Management program (IPM) is valuable for site specific pest management or for precise timing of targeting specific populations. The detection of spatial synchronization between the different pest populations may be useful for minimizing direct control tactics.

## Conclusions

The proposed multivariate modeling approaches provide a means to detect significant correlations and causality in ecological time-series. Regardless of the fact that correlations by themselves are crude, they may provide a novel basis for the study of non-trivial spatiotemporal relations in ecological time-series. The advantage of the Granger rules over correlations is that the Granger rules have dynamics features and thus provide an easy way to analyze the dynamic causal relations of a heterogeneous system of time-series variables.

In particular, causal measures overcome certain limitations in studying ecological relations because (i) they introduce a mathematical context and properties that serve as a core to construct network relations, (ii) they incorporate causal relations with a probabilistic and no subjective nature and (iii) they are closer to reality, because they not only incorporate the dynamics of all kinds of ecological variables but can be projected over the same landscape layer, allowing the development of inferences (e.g., populations evolve simultaneously with the surrounding environment).

Moreover, the Granger rules provide a means to detect synchronization and to detect which of the series may act as forcing variables. It is shown that populations of the same species are synchronized to a high degree, while populations of some locations exert a causal effect on others and may be used to predict the dynamics of the latter. The landscape-projected graphical models also describe the spatial patterns of causation and may be useful for site-specific management. The constructed networks may have practical utility because they suggest where to monitor populations to predict, for example, increases or declines of populations at other locations at the end of an edge in the graphs.

By extending these models, specifically by including more variables, it should be possible to explore interspecies and interspecies relationships with larger ecological systems and identify specific population traits that might constrain their structures in larger areas.

## Additional file

10.1186/s12898-016-0087-7 Actual ecological time series registered during successive growth seasons of the years 2003–2011 (3-day time intervals). W1: temperature (°C), W2: relative humidity (%), X1–X8: *A. orana*, Y1–Y3: *A. lineatella* and Z1–Z2: *G. molesta* moth populations (individuals).

## Abbreviations

FDR: false discovery rate
CROCO: cross correlations
PACO: partial cross correlations
MVAR: multivariate vector autoregression process
CGI: granger causality index
CGCI: conditional Granger causality index
ARMA: autoregressive moving average model
IPM: integrated pest management
